# Serum irisin and myostatin levels after 2 weeks of high-altitude climbing

**DOI:** 10.1371/journal.pone.0181259

**Published:** 2017-07-21

**Authors:** Ewa Śliwicka, Tomasz Cisoń, Zbigniew Kasprzak, Alicja Nowak, Łucja Pilaczyńska-Szcześniak

**Affiliations:** 1 Department of Hygiene, Poznań University of Physical Education, Poznań, Poland; 2 Department of Biomedical Sciences, State Higher Vocational School, Nowy Sącz, Poland; Universidad Pablo de Olavide, SPAIN

## Abstract

Exposure to high-altitude hypoxia causes physiological and metabolic adaptive changes by disturbing homeostasis. Hypoxia-related changes in skeletal muscle affect the closely interconnected energy and regeneration processes. The balance between protein synthesis and degradation in the skeletal muscle is regulated by several molecules such as myostatin, cytokines, vitamin D, and irisin. This study investigates changes in irisin and myostatin levels in male climbers after a 2-week high-altitude expedition, and their association with 25(OH)D and indices of inflammatory processes. The study was performed in 8 men aged between 23 and 31 years, who participated in a 2-week climbing expedition in the Alps. The measurements of body composition and serum concentrations of irisin, myostatin, 25(OH)D, interleukin-6, myoglobin, high-sensitivity C-reactive protein, osteoprotegerin, and high-sensitivity soluble receptor activator of NF-κB ligand (sRANKL) were performed before and after expedition. A 2-week exposure to hypobaric hypoxia caused significant decrease in body mass, body mass index (BMI), free fat mass and irisin, 25-Hydroxyvitamin D levels. On the other hand, significant increase in the levels of myoglobin, high-sensitivity C-reactive protein, interleukin-6, and osteoprotegerin were noted. The observed correlations of irisin with 25(OH)D levels, as well as myostatin levels with inflammatory markers and the OPG/RANKL ratio indicate that these myokines may be involved in the energy-related processes and skeletal muscle regeneration in response to 2-week exposure to hypobaric hypoxia.

## Introduction

Exposure to high-altitude hypoxia is a characteristic feature of mountain climbing. Numerous studies indicate that physical exercise at high altitudes causes physiological and metabolic adaptive changes by disturbing homeostasis [1,2]. Hypoxia-related changes in skeletal muscle affect the closely interconnected energy and regeneration processes [3,4,5]. Chronic hypoxia significantly potentiates the exercise-induced generation of reactive oxygen and nitrogen species and pro-inflammatory factors (TNFα, IL-1β, IL-6), which may in turn impair mitochondrial function and damage myocytes [6,7].

The altitude-induced shift in muscle protein turnover into tissue waste is followed by tissue regeneration processes. The balance between protein synthesis and degradation in the skeletal muscle is regulated by several molecules such as myostatin, cytokines, vitamin D, and irisin [8–12]. Myostatin is a transforming growth factor beta (TGF-β) that inhibits muscle cell growth by modulating Akt signaling pathways [5]. Its concentration may be reduced under the influence of calcitriol—the hormonally active metabolite of vitamin D—which regulates the expression of vitamin D receptors (VDR) in muscle cells and promotes their differentiation through the expression of insulin-like growth factor-II (IGF-II) and follistatin [13–15]. Irisin is a recently identified myokine that is released into the blood circulation during exercise. It plays an important role in the modification of energy processes, especially energy expenditure and glucose and lipid metabolism [10,16].

Shan et al. [17] suggested that myostatin is also involved in energy processes because it stimulates the muscle expression of irisin through the AMP-activated protein kinase-peroxisome proliferator-activated receptor coactivator 1α (PGC1α) pathway. Further, in their in vitro study, Vaughan et al. [18] showed that irisin activity in skeletal muscle increases energy expenditure and oxidative metabolism through the induction of PGC1α. Hypoxia significantly modifies systemic energy processes toward anaerobic processes [1]. Huh et al. [19] suggested that muscle mass was the primary predictor of circulating irisin underlying the association between irisin and metabolic factors. However, to our knowledge, no studies have examined the impact of physical exercise in hypobaric hypoxic conditions on irisin and myostatin levels.

The aim of the present study was to assess changes in irisin and myostatin levels in male climbers after a 2-week high-altitude expedition, and their association with 25(OH)D and indices of inflammatory processes.

## Materials and methods

### Study subjects and design

The study was conducted in 8 men aged between 23 and 31 years (mean ± SD: 27 ± 2.8 years), who participated in a climbing expedition in the Alps. All participants were physically active, experienced hikers and climbers. None of them had stayed at a high-altitude in the 6 months before the expedition. The study protocol was approved by the Ethics Committee for Human Research of the Poznań University of Medical Sciences. All participants in this study gave their written informed consent.

The expedition lasted 14 days, from late July to early August. The mountaineers began their ascent from 1000 m. In the first 24 hours, they reached an elevation of 3000 m and set up the first base camp, where they began a 24-hour acclimatization. No symptoms of acute mountain sickness were observed. The next day, they ascended to 3,800 m, and then returned to the base camp at 3,616 m. Every 2–3 days, they set up a base camp between 3,200 and 3,616 m. The ascent lasted from 6 to 16 hours a day, at a temperature of 14°C to −12°C. The participants’ intention was to climb 4000 m peaks in the Mont Blanc massif.

The day before their departure for the mountains and 2 days after returning to sea level, the participants were subjected to anthropometric and body composition measurements. Additionally, blood samples were collected for biochemical analyses.

### Anthropometric measures

Body mass and body composition (fat mass, free fat mass, were measured in the fasting state by using the Tanita BC-418MA Segmental Body Composition Analyzer (Tanita Corporation, Tokyo, Japan). The subjects stood barefoot on the analyzer and held a pair of handgrips, one in each hand. Their body height was measured using a mechanical measuring rod with an accuracy of 0.5 cm (WPT 60/150.O; Radwag, Radom, Poland). Body mass index (BMI) was calculated by dividing body mass (kg) by the square of body height (m^2^).

### Biochemical analyses

Blood samples were collected from the basilic vein after overnight fasting (between 8 a.m. and 9 a.m.) and centrifuged at 4000 rpm and 4°C. The serum was separated from the sample and stored at -70°C. High-sensitivity C-reactive protein (hsCRP) and myoglobin levels were determined using the immunoenzymatic method with commercially available kits (DRG International Inc., Springfield Township, NJ, USA; test sensitivity: 0.1 mg/L and 5 ng/mL, respectively), as were the levels of high-sensitivity IL-6 (R&D Systems Inc., Minneapolis, MN, USA; test sensitivity: 0.039 pg/mL), irisin (Aviscera Bioscience Inc., Santa Clara, CA, USA; test sensitivity: 100 pg/mL), osteoprotegerin (OPG) and high-sensitivity soluble receptor activator of NF-κB ligand (sRANKL) (Biomedica GmbH & Co KG, Wien, Austria; test sensitivity: 0.07 pmol/L and 0.01 pmol/L, respectively), myostatin (Immundiagostic AG, Bensheim, Germany; test sensitivity: 0.37 ng/mL), and 25(OH)D (Demeditec Diagnostic GmbH, Kiel, Germany; test sensitivity: 1.9 ng/mL).

### Statistical analysis

Data were presented as means, standard deviations (SD), medians (Me) and quartiles (Q_1_, Q_3_). The Shapiro-Wilk test was used to check the data for normal distribution. The values of normally distributed variables before the expedition were compared to those after the expedition using Student’s *t*-test, and non-normally distributed variables were similarly compared using the Wilcoxon test. The level of statistical significance was set at *P* < 0.05. The relationship between variables was determined using Spearman’s rank correlation. All analyses were performed using the Statistica 12.0 software package (StatSoft, Tulsa, Oklahoma, USA).

## Results

**Fig 1:**
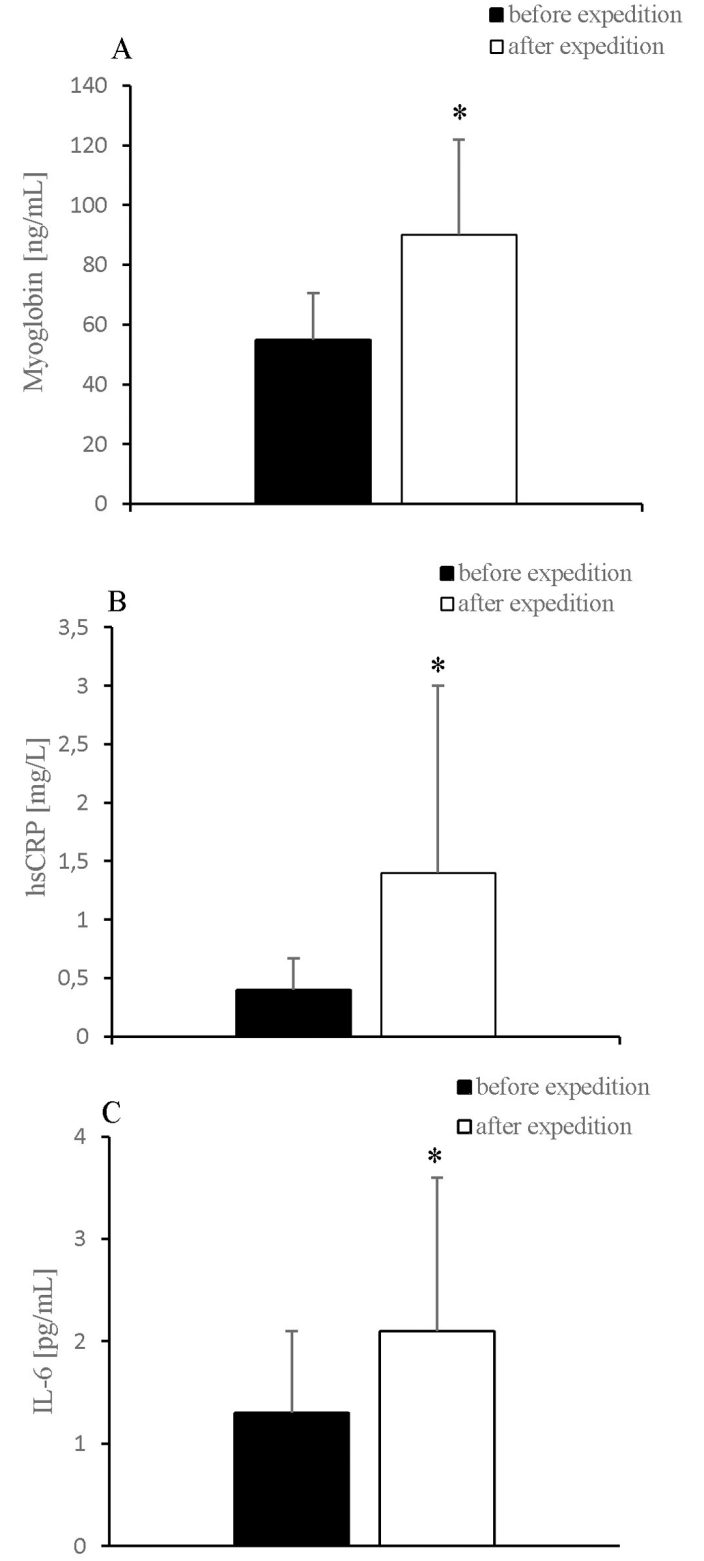
Changes in A: Myoglobin, B: hsCRP and C: IL-6 levels after 2-week exposure to hypobaric hypoxia. Values are means ± SD. * *P* < 0.05, significant change from baseline.

**Fig 2:**
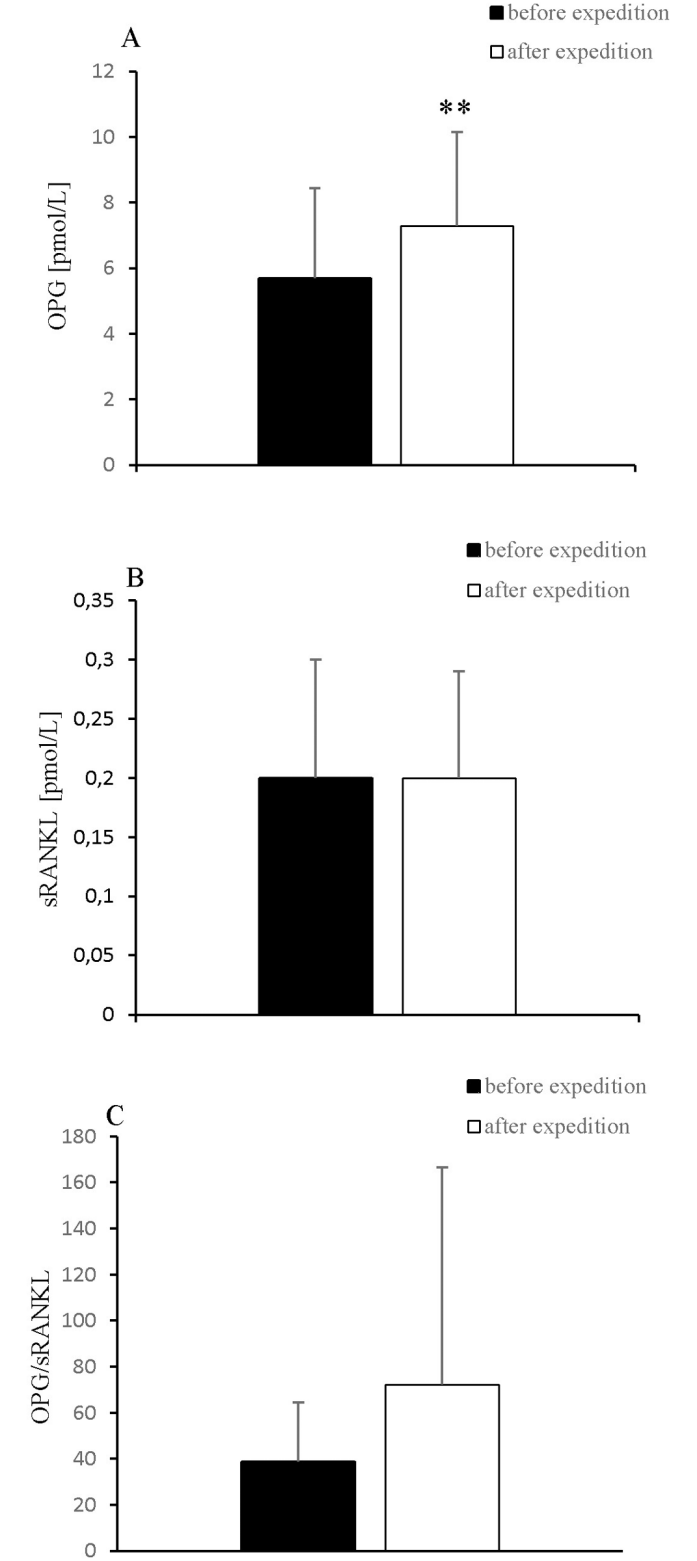
Changes in A: OPG, B: sRANKL levels and C: OPG/sRANKL ratio after 2-week exposure to hypobaric hypoxia. Values are means ± SD. * *P* < 0.05, significant change from baseline.

**Fig 3:**
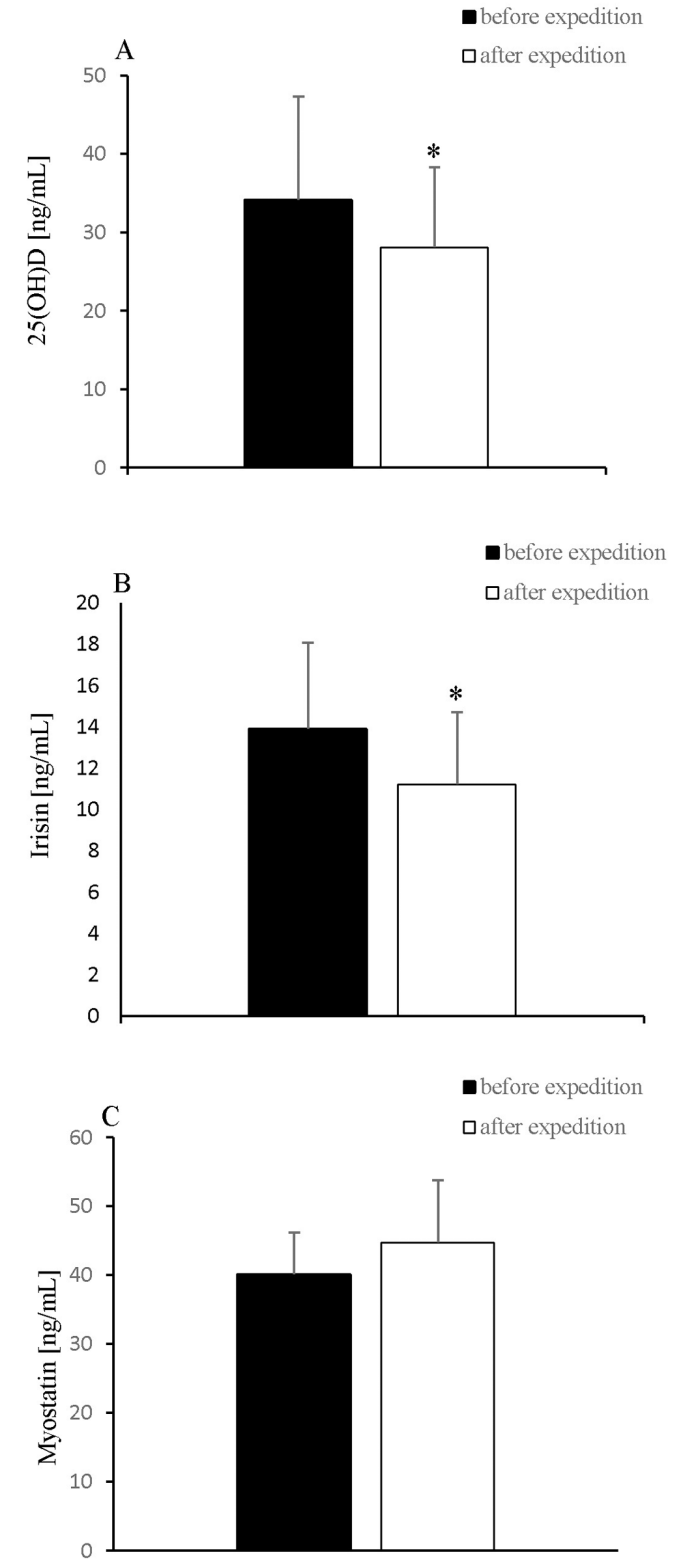
Changes in A: 25(OH)D, B: Irisin and C: Myostatin levels after 2-week exposure to hypobaric hypoxia. Values are means ± SD. * *P* < 0.05, significant change from baseline.

The participants’ body mass, BMI, and free fat mass decreased significantly after the expedition (Table 1). Further, 2-week exposure to hypobaric hypoxia caused a significant increase in the levels of myoglobin, hsCRP, hsIL-6 (Fig 1), and OPG (Fig 2A) and a significant decrease in the 25(OH)D and irisin levels (Fig 3A and 3B).

**Table 1:** Somatic parameters of the participants before and after the high-altitude climbing expedition.

Variable	Before expedition		After expedition		Δ [%]
**BM [kg]**	72.3±6.05	71.4 (67.9–78.2)	70.7±5.18	70.0 (66.8–74.5)	-2.2*
**BMI [kg/m**^**2**^**]**	22.7±1.27	22.7 (21.7–23.7)	22.2±0.83	22.2 (21.5–23.0)	-2.2*
**FFM [%]**	84.2±2.36	84.6 (83.5–85.8)	84.1±2.78	83.8 (83.4–86.2)	-0.1
**FFM [kg]**	60.9±5.80	60.6 (56.2–66.6)	59.5±5.13	58.7 (55.6–64.5)	-2.3*
**FM [%]**	15.8±2.36	15.5 (14.2–16.5)	15.9±2.78	16.2 (13.8–16.7)	+0.6
**FM [kg]**	11.4±1.61	11.6 (10.3–12.3)	11.2±1.89	11.3 (10.0–12.3)	-1.8
**Water [%]**	57.8±1.84	58.0 (57.3–59.1)	57.9±2.30	57.9 (56.6–59.7)	+0.2

Data are presented as mean ± SD and Me (Q_1_–Q_3_). BM, body mass; BMI, body mass index; FFM, fat free mass; FM, fat mass.

* *P* < 0.05, significant change from baseline.

The analysis showed significant correlations between the levels of irisin and lean body mass (%) before and after expedition (r = 0.92, *P* = 0.0009 and r = 0.74, *P* = 0.0366 respectively). After the expedition positive relationships were found between hsCRP and OPG levels (r = 0.71, *P* = 0.0465) and the OPG/sRANKL ratio (r = 0.83, *P* = 0.0102) as well as between levels of hsIL-6 and myostatin (r = 0.76, *P* = 0.0280) and irisin and 25(OH)D (r = 0.74, *P* = 0.0366). Positive correlations were also found between changes (Δ_I-II_) in the hsCRP and myostatin levels (r = 0.76, *P* = 0.0280), between hsCRP levels and the OPG/sRANKL ratio (r = 0.93, *P* = 0.0009), and between OPG/sRANKL and the myostatin levels (r = 0.86, *P* = 0.0065).

## Discussion

The results of the present study showed that exposure to hypobaric hypoxia (at 3000 m above sea level) for 2 weeks caused several changes in morphological and biochemical indices. First, the irisin and 25-Hydroxyvitamin D levels decreased significantly. The decrease in the irisin levels could be associated with a cascade of cellular energy metabolic responses leading to the shift from aerobic to anaerobic metabolism and a reduction in mitochondrial density. Zhang et al. [20] showed that hypoxia downregulates mitochondrial biogenesis factors such as PGC1α, which directly affects the expression of transmembrane protein fibronectin type-III domain containing protein 5, which is then cleaved and released in the circulation in the form of irisin [16].

Savkur et al. [21] suggested that PGC1α functions as a co-activator for VDR. Thus, the positive correlation between the 25(OH)D and irisin levels in the participants after the climbing expedition indicates the role played by vitamin D in PGC1α signaling pathways [21]. Moreover, Sinha et al. [22] found a significant relationship between vitamin D and mitochondrial function. The presence of VDR in mitochondria confirms the involvement of vitamin D in the regulation of aerobic processes, and the decrease in the 25(OH)D concentration resulting from hypoxia may be the mechanism by which these processes are inhibited.

As indicated in our earlier study, the reasons for the decrease in serum 25(OH)D levels on exposure to hypoxia could be the intensification of inflammatory processes [23]. Studies have shown that this vitamin may possess natural antioxidant and anti-inflammatory properties [24,25]. Intensified inflammation was also indicated by the increase in the serum hsCRP and hsIL-6 levels observed in the climbers in the present study, and our findings partially correspond with those of other studies [26–28].

Exposure to high-altitude hypoxia causes heterogeneous hypoxic pulmonary vasoconstriction, that leads to increase levels of inflammatory factors [29–31]. These factors are produced by endothelial cells, leucocytes, and damaged skeletal myocytes [32]. RANKL and its receptor RANK are critical regulators of immune responses [33], while OPG is a key player in suppressing the RANKL/RANK system and may work together with anti-inflammatory cytokines to inhibit inflammation [34,35]. In the present study, however, sRANKL levels showed no significant changes after the expedition despite a significant increase in the hsIL-6 and hsCRP levels. It might be assumed that these changes resulted from an increase in OPG, which inhibits the activity of the RANK/RANKL system.

In an animal model of muscular dystrophy, Dufresne et al. [36] found for the first time that muscle cells produce and secrete OPG, which play a critical role in controlling muscle function. Moreover, through their study on humans, Philippou et al. [37] suggested that the OPG/RANKL system may play an important role in muscle inflammation and repair, because they found a significant association between the serum levels of hsIL-6 and OPG and as well as the OPG/RANKL ratio after eccentric exercise. The present study found a positive correlation between hsCRP and OPG levels and the OPG/sRANKL ratio after the participants returned from the climbing expedition (the changes (Δ_I-II_) in hsCRP and OPG/sRANKL were also positively correlated). This may indicate the compensatory role played by OPG in inhibiting the inflammatory response by regulating the RANK/RANKL signaling pathway.

The positive correlation observed between the myostatin and hsIL-6 levels in the participants after they returned from the climbing expedition and between the changes (Δ_I-II_) in myostatin levels and the hsCRP levels and OPG/sRANKL ratio may indicate the role played by inflammatory factors in inhibiting the regeneration of damaged muscle tissue. Damage to the muscle fibers after the expedition is indicated by the significant increase in the myoglobin blood level, which may be caused by physical, mostly eccentric, exercise under hypoxic conditions. Our results correspond to those of other studies [38–40]. In fact, Philippou et al. [37] showed that the increased myoglobin levels after intense eccentric exercises persisted for several days.

Previous studies concluded that prolonged exposure to hypobaric hypoxia may reduce the cross-section of muscle fibers and muscle mass [41–44]. The climbers in the present study showed a significantly decreased lean body mass (kg) after the expedition, with no changes in the percent or absolute body fat mass. Similar observations were made by Sillau and Banchero [45] in healthy individuals living at high altitudes for generations. Further, in their animal model study, they found that muscle mass decreased following prolonged exposure to high-altitude hypoxia.

The reasons for the reduction in lean body mass include the energy deficit under hypoxic conditions [46], duration of exposure [47], and the energy expenditure during exercise [48]. In our view, this reduction may also be related to the decrease in the irisin and 25-Hydroxyvitamin D levels, since both irisin and 1,25(OH)_2_D, which is the hydroxylated metabolite of 25(OH)D, affect muscle regeneration by modulating energy processes [18,22,49]. The obtained correlations between irisin levels and lean body mass before and after the expedition are in line with results of the cross-sectional study on Korean population by Chang et al. [50]. The authors observed correlations between serum irisin concentrations and anthropometric parameters and suggested that this myokine may be a novel biomarker for sarcopenia.

## Conclusions

In summary, the observed correlations of irisin with 25(OH)D levels, as well as myostatin levels with inflammatory markers and the OPG/RANKL ratio indicate that these myokines may be involved in the energy-related processes and skeletal muscle regeneration in response to 2-week exposure to hypobaric hypoxia.

Although the results of this study indicate the importance of irisin and myostatin in muscle energy and regeneration processes, further research is needed to fully expound these findings. The small sample size was a constraining factor for this study, as well as fact that the blood tests were taken 2 days after participants had left the high altitude.

## Supporting information

S1 Appendix The dataset used in this study. (XLS)

S1 Fig Changes in myoglobin, hsCRP and IL-6 levels after 2-week exposure to hypobaric hypoxia. (XLS)

S2 Fig Changes in OPG, sRANKL levels and OPG/sRANKL ratio after 2-week exposure to hypobaric hypoxia. (XLS)

S3 Fig Changes in 25(OH)D, irisin and myostatin levels after 2-week exposure to hypobaric hypoxia. (XLS)
